# Assisted protein folding at low temperature: evolutionary adaptation of the Antarctic fish chaperonin CCT and its client proteins

**DOI:** 10.1242/bio.20147427

**Published:** 2014-03-19

**Authors:** Jorge Cuellar, Hugo Yébenes, Sandra K. Parker, Gerardo Carranza, Marina Serna, José María Valpuesta, Juan Carlos Zabala, H. William Detrich

**Affiliations:** 1 Centro Nacional de Biotechnología (CNB-CSIC), Campus de la Universidad Autónoma de Madrid, 28049 Madrid, Spain; 2 Department of Marine and Environmental Sciences and Department of Biology, Northeastern University, Marine Science Center, Nahant, MA 01908, USA; 3 Departamento de Biología Molecular, Universidad de Cantabria-IFIMAV, 39011 Santander, Spain; * Present address: Centro de Investigaciones Biológicas (CIB-CSIC), 28040 Madrid, Spain.

**Keywords:** CCT, TriC, Chaperone, Chaperonin, Protein folding, Actin, Tubulin, Thermal adaptation, Evolution

## Abstract

Eukaryotic ectotherms of the Southern Ocean face energetic challenges to protein folding assisted by the cytosolic chaperonin CCT. We hypothesize that CCT and its client proteins (CPs) have co-evolved molecular adaptations that facilitate CCT–CP interaction and the ATP-driven folding cycle at low temperature. To test this hypothesis, we compared the functional and structural properties of CCT–CP systems from testis tissues of an Antarctic fish, *Gobionotothen gibberifrons* (Lönnberg) (habitat/body T = −1.9 to +2°C), and of the cow (body T = 37°C). We examined the temperature dependence of the binding of denatured CPs (β-actin, β-tubulin) by fish and bovine CCTs, both in homologous and heterologous combinations and at temperatures between −4°C and 20°C, in a buffer conducive to binding of the denatured CP to the open conformation of CCT. In homologous combination, the percentage of *G. gibberifrons* CCT bound to CP declined linearly with increasing temperature, whereas the converse was true for bovine CCT. Binding of CCT to heterologous CPs was low, irrespective of temperature. When reactions were supplemented with ATP, *G. gibberifrons* CCT catalyzed the folding and release of actin at 2°C. The ATPase activity of apo-CCT from *G. gibberifrons* at 4°C was ∼2.5-fold greater than that of apo-bovine CCT, whereas equivalent activities were observed at 20°C. Based on these results, we conclude that the catalytic folding cycle of CCT from Antarctic fishes is partially compensated at their habitat temperature, probably by means of enhanced CP-binding affinity and increased flexibility of the CCT subunits.

## INTRODUCTION

Protein quality control and maintenance of the proteome are essential for the health of cells and organisms (Hartl et al., 2011). Most proteins must acquire precise, but flexible and minimally stable, three-dimensional structures to function within cells. Because the “folding landscape” is complex, with many potential nonfunctional outcomes, cells produce molecular chaperones to guide efficient folding while preventing protein aggregation. Proteins that are irreversibly misfolded or aggregated are removed by the ubiquitin–proteasome system or by lysosomal autophagy (Ciechanover, 1998; Arias and Cuervo, 2011).

The chaperonin containing *t*-complex polypeptide-1 [CCT, aka TCP-1 ring complex (TriC)] plays a central role in cellular homeostasis by assisting the folding of ∼10% of newly synthesized proteins, including tubulins and actins (“client proteins” or CPs) (Thulasiraman et al., 1999; Valpuesta et al., 2002; Dekker et al., 2008; Yam et al., 2008; Valpuesta et al., 2005). CCT is a cylindrical, 16-subunit toroid composed of eight distinct subunits (CCTα–CCTθ) that form two eight subunit, back-to-back rings, each containing a folding “cage” for CPs (Yébenes et al., 2011; Leitner et al., 2012). Sequestration of CPs by CCT in a closed conformation and CP release require ATP binding, hydrolysis, and associated intra- and inter-ring allosteric signaling (Yébenes et al., 2011; Leitner et al., 2012; Cong et al., 2012). In some cases, additional protein co-factors are required either to deliver CPs to CCT or to facilitate final maturation and oligomerization of CPs after their interactions with CCT (Valpuesta et al., 2002; Valpuesta et al., 2005; Yébenes et al., 2011).

The Antarctic notothenioids are a unique, cold-adapted fish fauna whose evolution has been driven by the development of extreme low temperatures as the Southern Ocean cooled to the modern range, −1.9 to +2°C, over the past 25–40 million years (DeWitt, 1971; Lawver et al., 1992; Lawver et al., 1991; Eastman, 1993; Eastman and Clarke, 1998; Scher and Martin, 2006; DeConto and Pollard, 2003). The acquisition of novel antifreeze proteins by the notothenioids (Chen et al., 1997; Cheng and Chen, 1999), their evolution of a cold-stable microtubule cytoskeleton (Detrich et al., 1989; Detrich et al., 2000; Redeker et al., 2004), their loss of an inducible heat shock protein (HSP) response (Hofmann et al., 2000; Buckley et al., 2004; Detrich et al., 2012), and the loss of hemoglobin expression by the icefish family (Cocca et al., 1995; Near et al., 2006; Zhao et al., 1998) are examples of novel traits that evolved over 5–10 million years of isolation in a perennially icy environment. Today, these stenothermal fishes are threatened by rapid warming of the Southern Ocean (∼1–2°C per century) over periods measured in centuries or less (Gille, 2002; Clarke et al., 2007; Ducklow et al., 2007; Pörtner et al., 2007; Pörtner and Farrell, 2008; Somero, 2005), which may challenge their capacity to maintain protein homeostasis.

Although High Antarctic notothenioids lack an inducible HSP response (Hofmann et al., 2000; Buckley et al., 2004; Buckley and Somero, 2009; Thorne et al., 2010), they do express constitutively many chaperones (Place et al., 2004; Place and Hofmann, 2005), including CCT (Pucciarelli et al., 2006), very likely to counteract the seemingly paradoxical cold-induced denaturation of proteins (Todgham et al., 2007; Lopez et al., 2008; Dias et al., 2010). Thus, we hypothesize that Antarctic notothenioids have evolved a chaperonin that is compensated, at least in part, to maintain folding activity at low temperature. To test this hypothesis, we have purified CCT from testis tissue of the Antarctic Humphead notothen, *Gobionotothen gibberifrons* (Lönnberg), and have compared its structural and functional properties to those of bovine testis CCT. The apparent affinity of *G. gibberifrons* CCT for homologous CPs is high at low temperature and declines as temperature increases, whereas the opposite behavior was observed for bovine CCT and CPs; affinity increased with increasing temperature. Furthermore, the ATPase activity of apo-CCT from the Antarctic fish is substantially greater at cold temperature than that of bovine CCT. We suggest that adaptation of the function of Antarctic fish CCT at low temperature is based on lowering the activation energy barrier(s) of the folding cycle through enhanced CP-binding affinity and increased subunit flexibility. Nevertheless, the thermal scope of the activity of *G. gibberifrons* CCT appears to be sufficient to tolerate temperatures as much as 5°C above their present habitat norm.

## RESULTS

### Purification of *G. gibberifrons* CCT

**Fig. 1.**
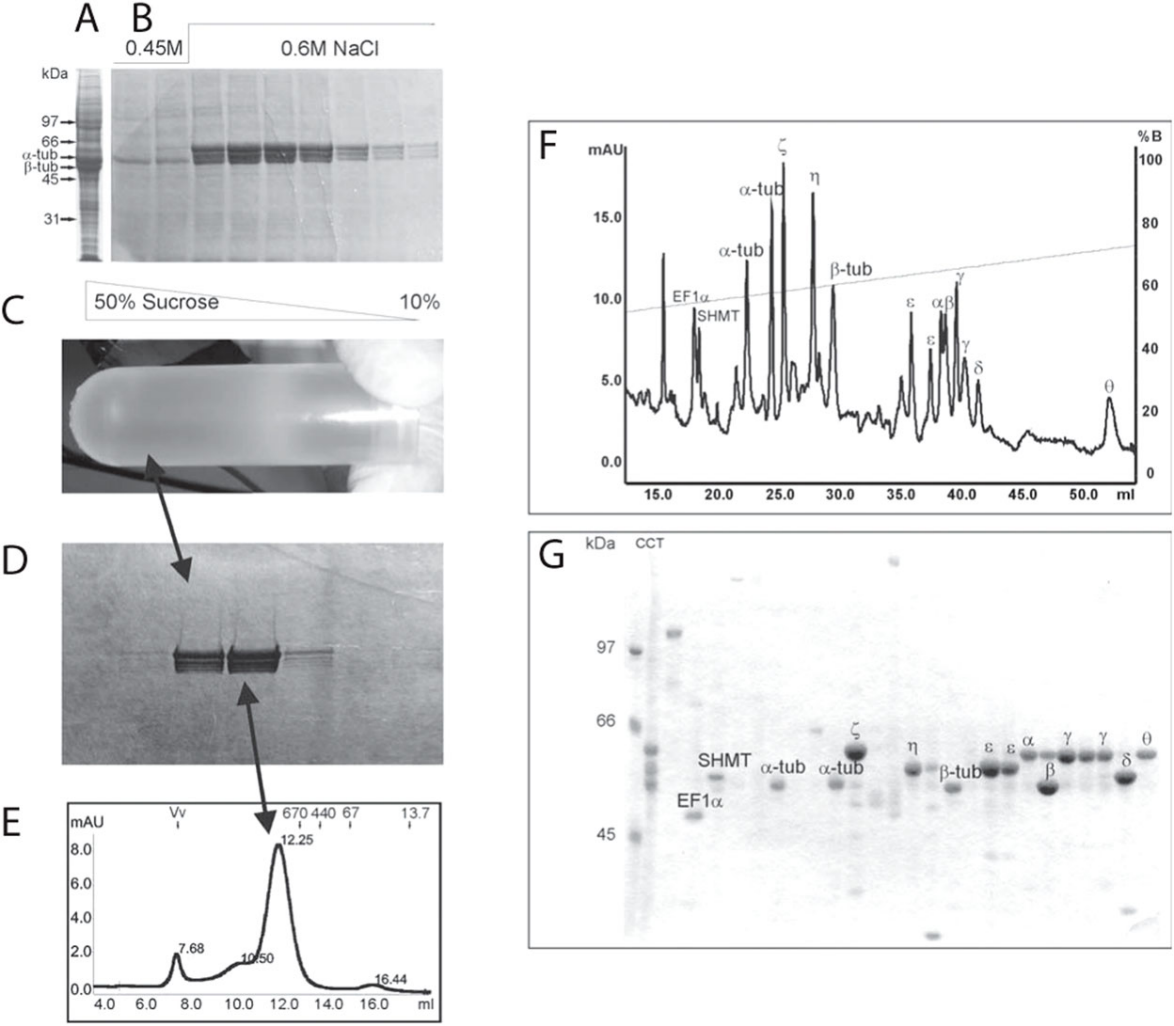
Purification of *G. gibberifrons* CCT from immature testis tissue and separation of subunits by HPLC. An ammonium sulfate cut (30–50%) of a centrifugal extract of testis tissue was chromatographed on Heparin Sepharose, CCT-containing fractions were pooled and centrifuged on sucrose gradients, and the ∼25S chaperonin fraction was chromatographed on Superose 6 (see Materials and Methods for details). The subunits of CCT were isolated by HPLC for subsequent analysis by mass spectrometry (Fig. 2). Throughout the purification, protein compositions of fractions were analyzed by SDS-PAGE. (A) Testis extract, dominated by α- and β-tubulins, prior to application to Heparin Sepharose. (B) Fractions containing CCT (subunits ∼55–60 kDa) eluted from the Heparin Sepharose column by a 0.45→0.6 M NaCl step gradient. (C,D) CCT-enriched fractions from Heparin chromatography (B) were pooled and then centrifuged through 10–50% sucrose gradients (C), and proteins sedimenting at ∼25 S were analyzed by electrophoresis (D). (E) Pooled CCT was loaded on a Superose 6 gel filtration column, and the material eluting at an M_r_ of ∼10^6^, which consisted of nearly homogeneous CCT, was collected. (F) HPLC elution profile of CCT subunits and several contaminating proteins; the starting material corresponded to purified CCT shown in Fig. 1D. (G) SDS-PAGE of HPLC fractions on 8.5% gels. Protein identities were established by mass spectrometry (Fig. 2): α–θ, CCT subunits; EF1α, elongation factor 1α; SHMT, serine hydroxymethyltransferase; α-tub, β-tub, α- and β-tubulins, respectively. Absorbance (mAU) and the solvent gradient (%B) are plotted *vs* elution volume in panel F. Note that CCT subunits γ and ε were each resolved as two peaks.

**Fig. 2.**
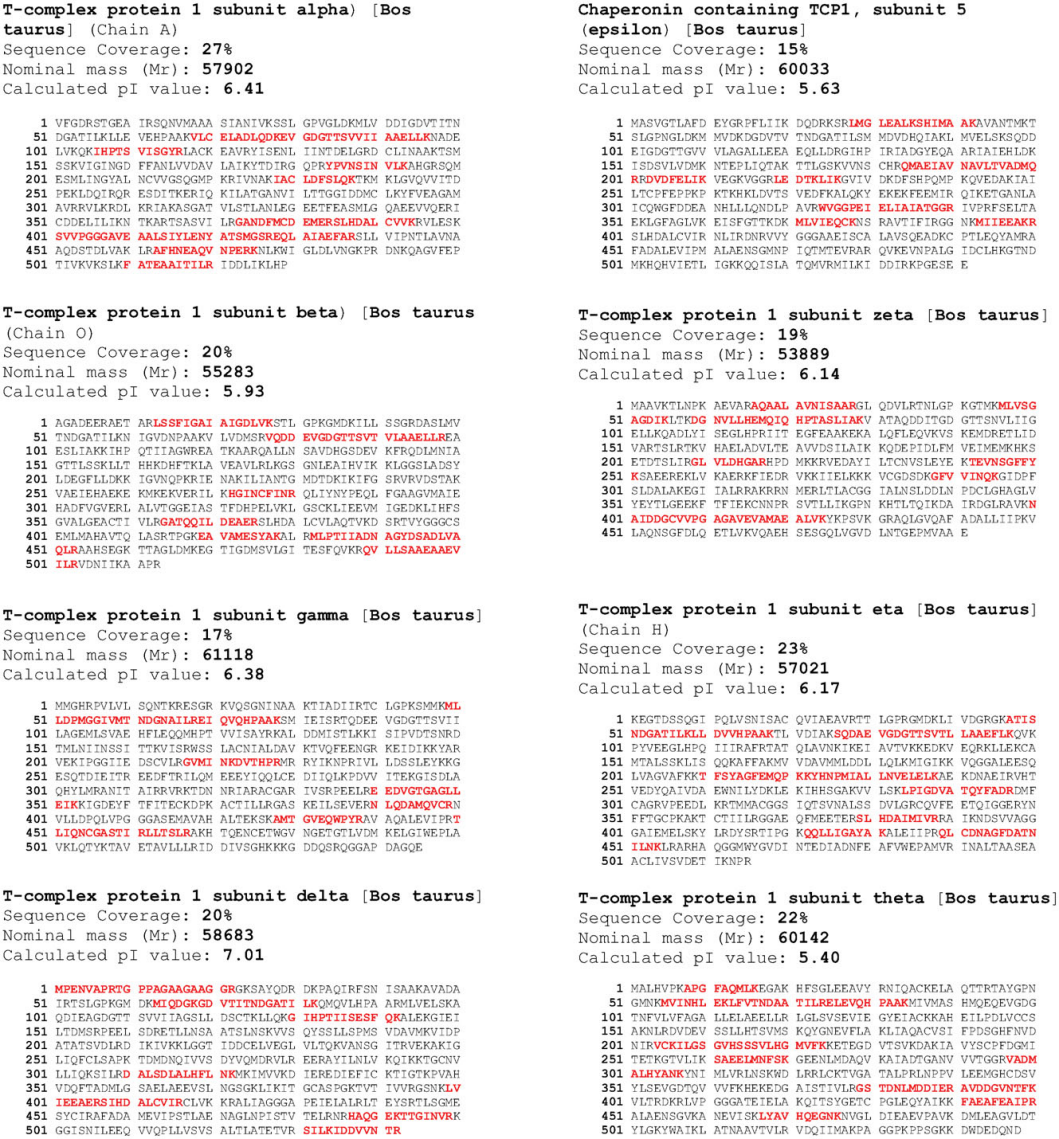
Identification of *G. gibberifrons* CCT subunits. Plugs containing the indicated protein bands were excised from the gel shown in Fig. 1G for in-gel tryptic proteolysis and mass spectrometric analysis. The identities of the presumptive *G. gibberifrons* CCT subunits were confirmed by querying the non-redundant NCBI protein database with the *G. gibberifrons* tryptic peptide sets. Each subunit possessed six, seven, or eight peptides that mapped perfectly to peptides of a bovine CCT subunit (red). Peptide sequence coverage ranged from 15–27% for the *G. gibberifrons*/bovine comparison, and higher values were found for comparison to CCT subunits from other fishes (data not shown). Percent coverage was highest for the β (67%) and θ (55%) subunits of CCT from the Antarctic Bullhead notothen, *N. coriiceps*, whose sequences had been established previously from cloned cDNAs (Pucciarelli et al., 2006). The calculated pIs correspond to the bovine CCT subunits.

The eukaryotic class-II chaperonin CCT possesses biochemical and biophysical properties – subunit size and heterogeneity, oligomeric structure, etc. – that can be exploited to purify the complex from diverse sources. Here we employed ion-exchange chromatography, sucrose-gradient ultracentrifugation, and size-exclusion chromatography to isolate CCT from immature testis tissue of the Antarctic Humphead notothen, *G. gibberifrons*. Fig. 1 shows the purification of *G. gibberifrons* CCT at several stages: 1) a 30–50% ammonium sulfate cut of a testis high-speed centrifugal extract, which, after dialysis, was applied to a Heparin Sepharose column (Fig. 1A); 2) the elution of bound chaperonin from Heparin Sepharose (Fig. 1B) by application of a step gradient of NaCl (0.45→0.6 M); 3) the banding position of the ∼25S CCT complex on a sucrose gradient (Fig. 1C); 4) its subunit complexity as revealed by SDS-PAGE (Fig. 1D); and 5) elution of CCT from a Superose 6 column at an apparent molecular weight of ∼1000 kDa (Fig. 1E). The Superose-6-purified CCT is nearly homogeneous and is composed of multiple subunits of M_r_ ∼55–60 kDa (Fig. 1D). The yield of CCT was 40 µg/g testis tissue, ∼4-fold greater than obtained by our previous method (Pucciarelli et al., 2006).

### CCT subunit identification and biochemical characterization

Using HPLC and mass spectrometry, we confirmed that *G. gibberifrons* CCT contained the eight canonical subunits (α, β, γ, δ, ε, ζ−1, η, θ) characteristic of the vertebrate chaperonin (Fig. 1F,G, Fig. 2); the mammalian ζ−2 variant was not detected. The pIs of five of the *G. gibberifrons* subunits were more basic than their bovine orthologs, and three had pIs that were more acidic (Table 1). The differences in pIs were generally concordant with compositional variation in charged amino acid residues (data not shown).

**Table 1.**
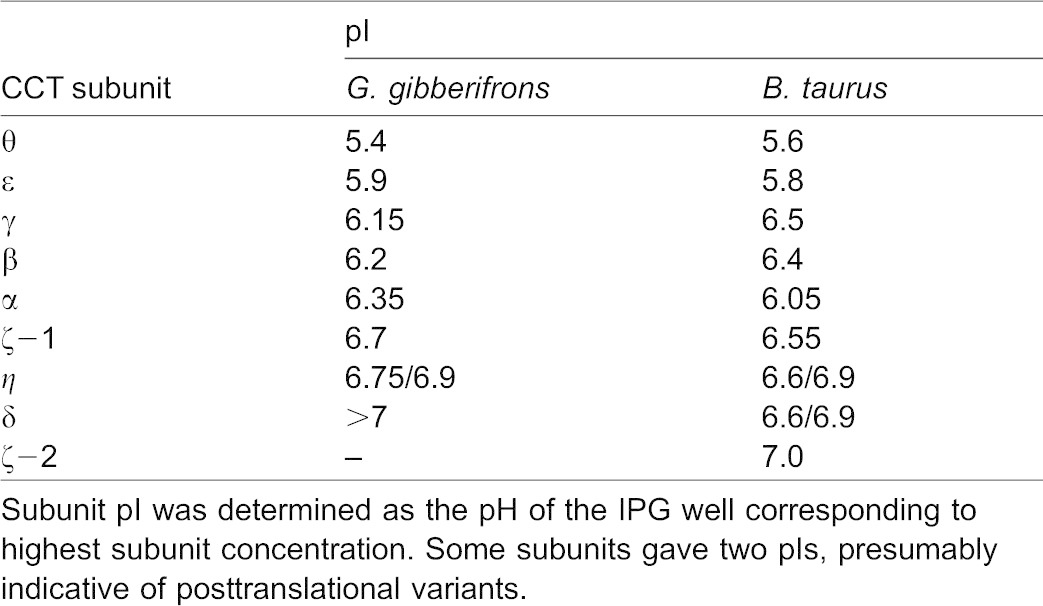
Isoelectric points of CCT subunits from an Antarctic fish and a mammal

Muñoz et al. have shown that many subunits of bovine testis CCT are posttranslationally modified by small, charged moieties (thought to be acetate and phosphate groups) to give multiple modified variants (Muñoz et al., 2011), and the mouse CCT subunits β, γ, δ, ε, ζ, and η contain multiple acetylated and/or phosphorylated residues (UniProtKB and references therein). By contrast, CCT subunits from *G. gibberifrons* were generally homogeneous in isoelectric point, with the exception of the η chain (Table 1). [Although the γ and ε chains of *G. gibberifrons* each eluted as two peaks from the C4 Reversed-Phase HPLC column (Fig. 1F,G), they did not show evidence of isoelectric heterogeneity (Table 1).] Substantial isoelectric heterogeneity was observed for bovine CCT subunits β, γ, δ, ε, ζ−1, and η (data not shown), consistent with the results of Muñoz et al. (Muñoz et al., 2011) and in agreement with the mouse data. Therefore, CCTs expressed by the Antarctic notothenioids appear to be modified to a lesser extent than are mammalian CCTs, an observation that mirrors the reduced polyglutamylation of notothenioid tubulins (Redeker et al., 2004).

### Structural characterization of *G. gibberifrons* CCT

**Fig. 3.**
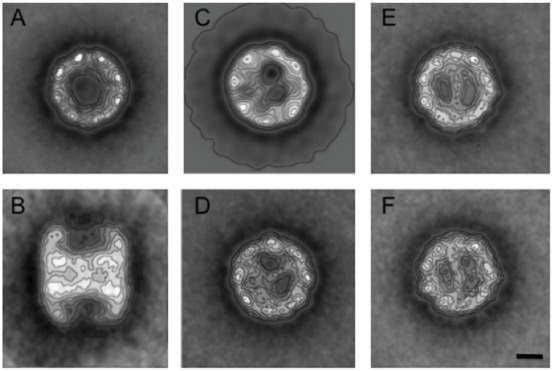
Structural characterization of *G. gibberifrons* CCT by EM: comparison to the bovine chaperonin. Two-dimensional average images of apo- and holo-CCTs were generated as described in Materials and Methods. (A) apo-CCT from *G. gibberifrons*, top view (*n* = 575 particles). (B) apo-CCT from *G. gibberifrons*, side view (*n* = 486 particles). (C) *G. gibberifrons* CCT in complex with *N. coriiceps* β1-tubulin, top view (*n* = 650 particles). (D) CCT–tubulin complex from the cow, top view (*n* = 570 particles). (E) *G. gibberifrons* CCT in complex with *C. aceratus* actin, top view (*n* = 710 particles). (F) CCT–actin complex from the cow, top view (*n* = 657 particles). Scale bar: 5 nm.

Negative-stain EM and image averaging of the notothen apo-chaperonin at 4°C showed that it conforms to the classical end-on and side views of eukaryotic CCT – a toroid composed of two eight-subunit rings (Fig. 3A) in back-to-back orientation (Fig. 3B). Each ring contains a binding “cage” for CPs (Yébenes et al., 2011; Leitner et al., 2012). When *G. gibberifrons* apo-CCT was incubated either with denatured β-tubulin or with denatured β-actin, the binding cage was found to be occupied by a stain-excluding mass that crossed the cavity (Fig. 3C,E, respectively) in a CP-specific arrangement that was virtually identical to that observed with bovine CCT and its orthologous CP (Fig. 3D,F, respectively) at 25°C. [Note that the α and β chains of the bovine tubulin CP bind identically to CCT (Llorca et al., 2000).] Therefore, the structures of CCT–CP complexes from a psychrophilic fish are quite similar to those previously reported for CCT–CP complexes from mesophilic species, an observation consistent with the general structural conservation of chaperonins and their clients in organisms adapted to distinct thermal regimes.

### Folding activity of *G. gibberifrons* CCT

**Fig. 4.**
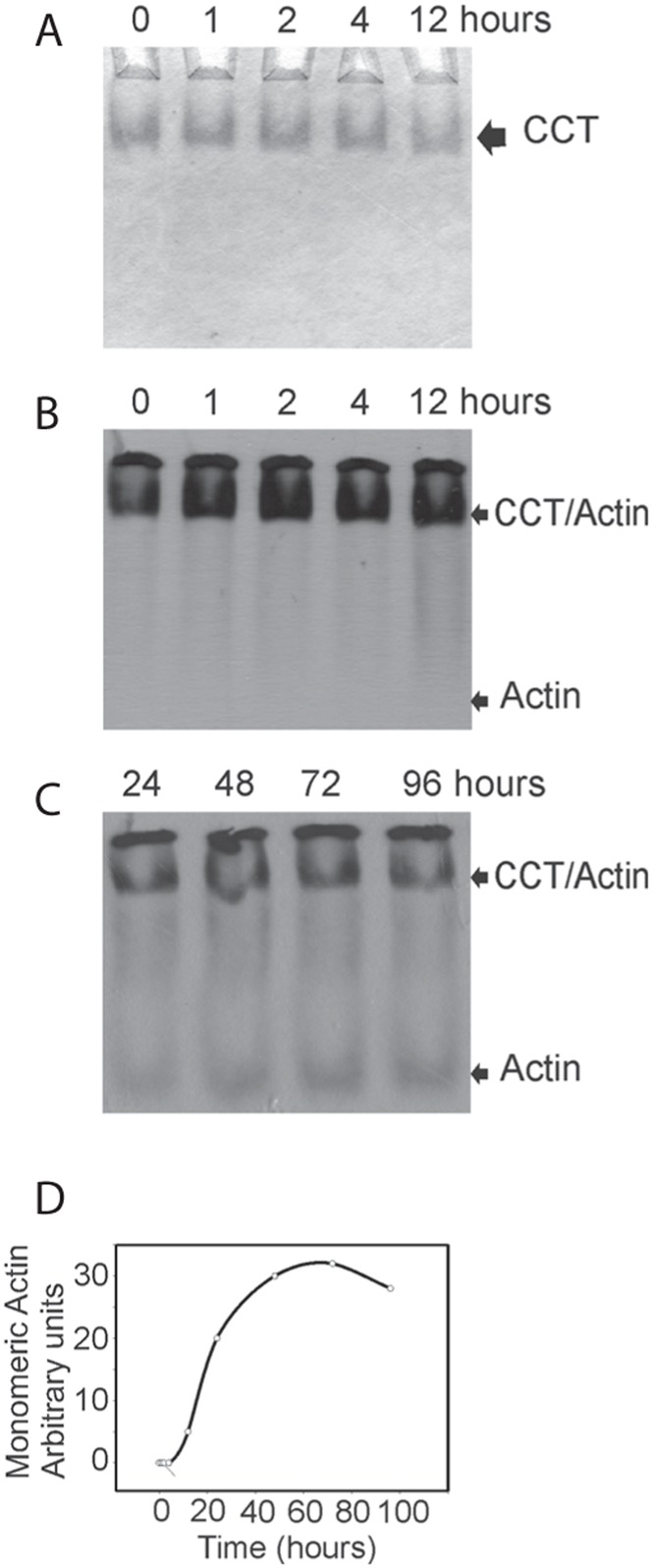
*G. gibberifrons* CCT binds to, folds, and releases *C. aceratus* actin at physiological temperature. CCT was incubated with denatured actin at intervals from 0 to 96 h at 2°C in binding buffer containing 1 mM Mg^2+^-ATP. Reaction products were analyzed at 2°C by non-denaturing electrophoresis on 4.5% polyacrylamide gels followed by autoradiography. (A) apo-CCT migrates as a single band as shown on this Coomassie Blue-stained gel. (B–D) Folded β-actin is detected at 12 h and increases in amount until a plateau is reached at 72–96 h. Large amounts of β-actin remained in complex with CCT. The positions of apo-CCT, of CCT–β-actin, and of folded actin monomer are indicated.

The hallmark of chaperonin function is the capacity to assist the folding of its denatured clients. To assess the folding activity of *G. gibberifrons* CCT at an environmentally relevant temperature, we incubated the chaperonin with ^35^S-labeled *C. aceratus* β-actin at 2°C and then added ATP. Fig. 4 shows that *G. gibberifrons* CCT was able to bind denatured β-actin and release it in its native conformation. Release of the folded product first became apparent at 12 h (Fig. 4B), after which accumulation followed a sigmoidal path to a plateau attained at ∼72 h (Fig. 4C,D). Thus, the folding cycle of Antarctic fish CCT at a temperature close to the physiological norm is considerably slower than that of mammalian CCT at 30°C (cf. Melki and Cowan, 1994), but this difference may simply be due to thermal scaling of the temperature coefficient, *Q_10_*. (Note that *Q_10_* for the ATPase activity of *G. gibberifrons* apoCCT scales normally between 4 and 20°C; see *ATPase activities of apoCCTs* below.) The failure to observe folding of actin at 4 or 20°C in our previous study of *N. coriiceps* CCT (Pucciarelli et al., 2006) probably resulted from the short incubation time (90 min) used in those assays.

### Temperature dependence of CP-binding by *G. gibberifrons* and bovine CCTs

**Fig. 5.**
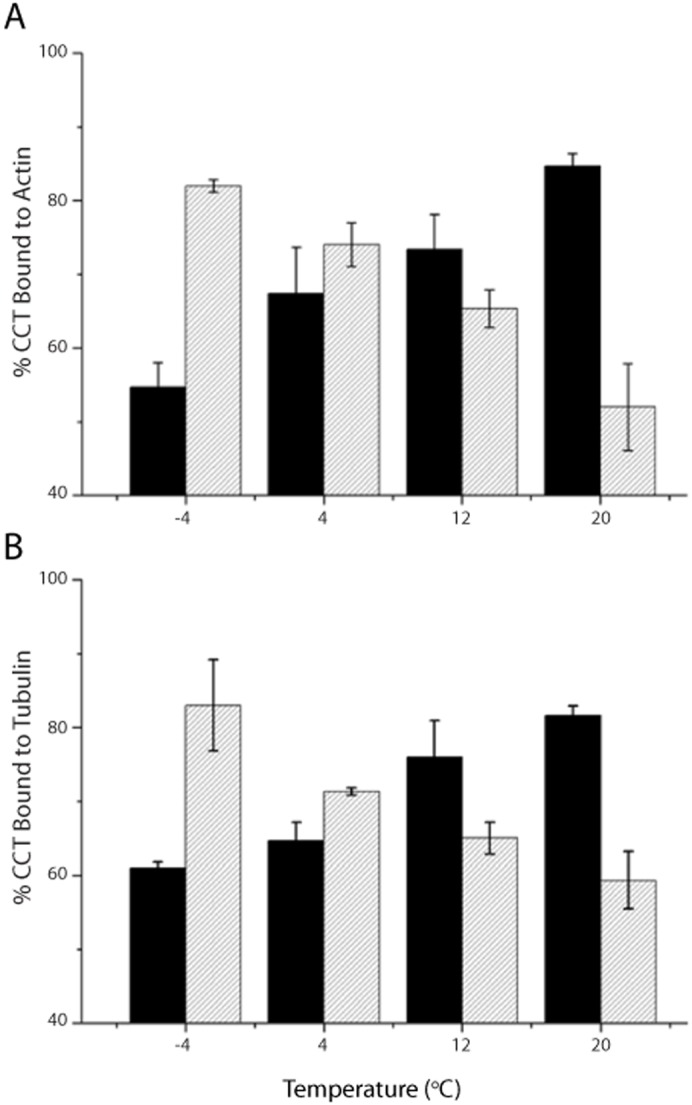
Temperature dependence of CP binding by testis CCTs from an Antarctic notothen and the cow. The binding of CPs by their homologous chaperonins was examined at four temperatures between −4°C and +20°C. Experiments were performed in triplicate, and at least 2000 top-view CCT particles from each binding reaction were scored automatically as apo- or holo-CCT as described in Materials and Methods. Data are presented as percentage CCT bound to CP (mean ± s.d.). *In toto*, >110,000 particles were scored. Client proteins: (A) actin; (B) tubulin. Chaperonins: *hatched bars*, *G. gibberifrons*; *black bars*, *Bos taurus* (cow).

Given the dramatic effects of temperature change on the kinetics and energetics of biochemical reactions, we hypothesize that the CCT and CPs of Antarctic fishes co-evolved to give productive substrate folding in the cold and, therefore, may not perform efficiently at elevated temperature. Conversely, we predict that bovine CCT and CPs would interact more effectively at elevated temperatures near the body temperatures of mammals (+37°C). We tested these predictions by comparing the binding of homologous combinations of CCT and CPs (actins, tubulins) at temperatures between −4°C and +20°C by electron microscopy and image processing. Fig. 5A shows the binding of Antarctic fish β-actin to the open conformation of *G. gibberifrons* CCT (*hatched bars*) *vs* the binding the bovine cardiac actin to bovine CCT (*black bars*); Fig. 5B presents comparable analyses with homologous tubulins as the CPs. The trends in the data are clear – irrespective of CP, the percentage of *G. gibberifrons* CCT that bound client declined with increasing temperature, whereas the percentage of bovine CCT bound to CP increased. The data were well fit by linear regression (Pearson's adjusted coefficient of determination, *R^2^*, ≥0.95 for the four fits; data not shown), which indicates that the binding percentages were very likely determined by the assay temperature. Our results are consistent with the co-evolution of the interaction surfaces of CCT and CPs from Antarctic fishes to yield high binding affinities at low temperature.

**Fig. 6.**
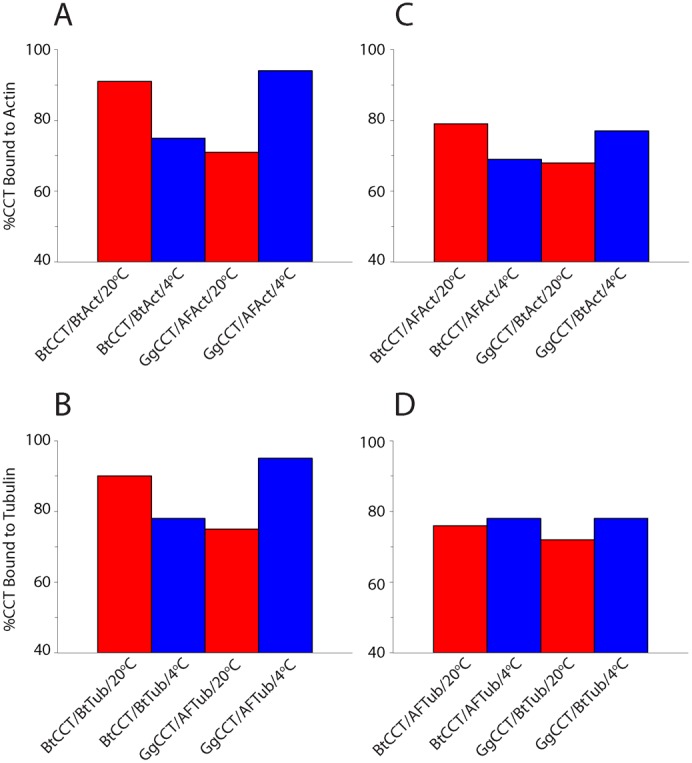
Temperature dependence of CP binding by CCT in homologous and heterologous combinations. CCT–CP binding reactions were performed and analyzed as described in Materials and Methods. (A,B) Homologous combinations of CCT and CP: actin (A); tubulin (B). (C,D) Heterologous combinations of CCT and CP: actin (C); tubulin (D). Incubation temperatures are given beneath each bar. *Red bars*, incubations performed at 20°C; *blue bars*, incubations performed at 4°C. Data are presented as percentage CCT bound to CP. Abbreviations: Act, actin; AF, Antarctic fish; Bt, *B. taurus*; Gg, *G. gibberifrons* (notothen); Tub, tubulin.

The analyses of the temperature dependence of the CCT–CP interaction in homologous combination suggest that the binding affinities of psychrophilic and mesophilic CCTs and CPs in heterologous combination would likely be low. Fig. 6 compares the binding of homologous and heterologous combinations of CCT and CPs at two temperatures, +4°C and +20°C. In homologous combinations (Fig. 6A,B), the results recapitulate those of Fig. 5A,B – the *G. gibberifrons* CCT–CP interaction was stronger at low temperature for both actin (A) and tubulin (B), whereas the converse was true for the bovine system. In heterologous combination (Fig. 6C,D), by contrast, CCTs and CPs interacted at lower affinity regardless of temperature. [The apparent affinities observed in Fig. 6 were on average 15% greater than comparable values reported in Fig. 5. This disparity may be attributed to small variations in CCT preparations, which were made on multiple occasions over several years. Although the experiments cannot be directly compared numerically, the same *patterns* of temperature dependence of CCT binding affinity emerge from the two data sets.] There was an indication that the CCTs bind either actin substrate with greater affinity at the “physiological” temperature of the chaperonin (Fig. 6C), but this trend was not seen for tubulin substrates (Fig. 6D). Because binding of the heterologous pairings was analyzed in a single experiment, the results must be interpreted cautiously.

### ATPase activities of apoCCTs

CCT possesses an intrinsic ATPase activity in the absence or presence of client proteins, and free energy released during the hydrolytic cycle drives the conformational cycle of the folding complex (Melki and Cowan, 1994). To determine whether CCT from *G. gibberifrons* exhibits thermal compensation of folding in the cold habitat experienced by the species, we compared its steady-state ATPase activities at psychrophilic and mesophilic temperatures to those of bovine CCT. Table 2 shows the ATPase activities of the two apoCCTs at 4 and 20°C, measured via a coupled-enzyme assay under conditions in which the concentration of CCT was the rate-limiting factor. At these temperatures, the ATPase activity of each CCT was linear for intervals ≥60 min, which indicates that neither the psychrophilic nor the mesophilic chaperonin denatured measurably during the assays. At 4°C, the ATPase activity of the notothen CCT was 2.6-fold greater than that of the bovine chaperonin, whereas the activities of the two CCTs were nearly identical at 20°C. The temperature coefficient, *Q_10_*, for *G. gibberifrons* CCT was 2.6, close to the range of 2.0–2.5 that typifies biochemical reactions involving protein conformational changes at physiological body temperatures (Hochachka and Somero, 2002). This “normal” *Q_10_* is somewhat surprising considering that 20°C is distinctly outside the habitat temperature range of the Antarctic fish, but many psychrophilic enzymes and structural proteins have activity optima at temperatures near 20–30°C (cf. D'Amico et al., 2003; Detrich et al., 2000; Detrich et al., 1992). By contrast, *Q_10_* for bovine CCT was 5.0, which is consistent with a steep decline in catalytic performance with decreasing temperature. We conclude that *G. gibberifrons* CCT is at least partially compensated for the rate-depressing effects of low temperature and is sufficiently stable to retain catalytic activity at mesophilic temperature, whereas the activity of the bovine chaperonin is significantly compromised at psychrophilic temperature.

**Table 2.**
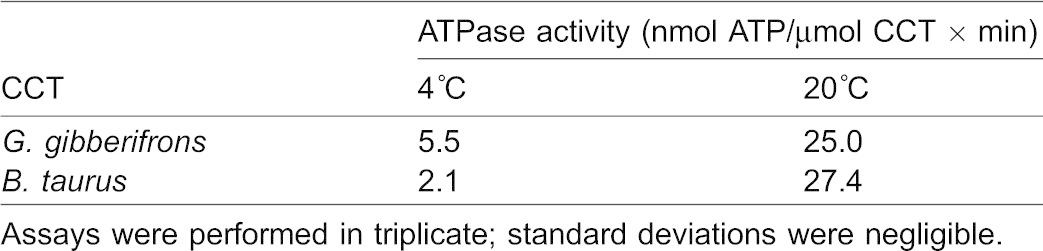
Temperature dependence of the ATPase activities of apoCCTs from an Antarctic fish and a mammal

## DISCUSSION

The successful radiation of Antarctic notothenioid fishes in the Southern Ocean involved constraints and trade-offs at many levels of biological organization: molecular, cellular, organismal, and ecological (Pörtner et al., 2007). At the molecular and cellular levels, numerous studies have documented compensatory thermal adaptation of individual proteins of these fishes relative to cool temperate notothenioids from South America and New Zealand and to temperate fishes in general (for reviews, see Coppes Petricorena and Somero, 2007; Somero, 2004). One may plausibly argue that widespread, cold-adaptive alteration of enzymes and structural proteins in the stenothermal Antarctic notothenioids would be disadvantageous, or perhaps lethal, should these fishes encounter the rapidly rising oceanic temperatures projected along the Antarctic Peninsula during the next century (Gille, 2002; Clarke et al., 2007; Ducklow et al., 2007; Pörtner et al., 2007). Thus, Somero's question – “how many proteins ‘need’ to adapt (in ectotherms) when temperature rises by a few degrees?” (Somero, 2011) – requires an expansive functional analysis of proteins belonging to many structural classes and different groups of organisms (Lockwood and Somero, 2012). Must a few proteins evolve, many, or a lot?

To evaluate the thermal tolerance of the notothenioid proteome to a warming marine environment, we have chosen to focus on the gatekeeper of cellular protein homeostasis, the cytoplasmic chaperonin CCT. This protein complex, which assists the folding of a large number of proteins of multiple structural classes and complex topologies (Thulasiraman et al., 1999; Dekker et al., 2008; Yam et al., 2008), could constitute a metabolic bottleneck in Antarctic notothenioid cells should its function be compromised by elevated temperature. Alternatively, retention of the capacity of notothenioid CCT to bind CPs and to assist their three-dimensional maturation at 5°C above the current habitat temperature of this fish group would support conjectures that a relatively small number of proteins might require adaptive fine-tuning of function and stability in the context of anticipated climate change (Somero, 2011; Powers and Schulte, 1998; Ream et al., 2003; Lockwood and Somero, 2012). Our results are consistent with the latter possibility, since the psychrophilic CCT appears to be folding-competent, and at least some of its CPs (e.g. tubulins) stable and active (Detrich et al., 1989; Detrich et al., 1992), at temperatures 5–10°C above the physiological.

The apparent affinity of *G. gibberifrons* testis CCT for its homologous actin and tubulin substrates at temperatures between −4°C and +4°C approximates the affinity of bovine testis CCT for its CPs at 20°C (Fig. 5). Since these temperatures are reasonable proxies for their respective cellular environments, our results imply that the two chaperonins have evolved corresponding states of the conformational flexibility necessary for binding and release of their CPs, and perhaps for the protein folding cycle as a whole. How might this be achieved? Comparative analyses of orthologous psychrophilic, mesophilic, and thermophilic enzymes have shown that the rate-limiting step in enzymatic catalysis is the flexibility of loops that must move to accommodate substrate binding and product release during the catalytic cycle (reviewed by Somero, 2004). For psychrophilic enzymes, amino acid substitutions that facilitate the flexibility of the hinge regions about which loops or domains must move are the key adaptive changes that lower activation energy barriers for catalytically critical conformational changes. Therefore, we propose that adaptive evolution of CCT for efficient function at psychrophilic temperatures may be based upon flexibility-enhancing residue substitutions in the apical lid, which appears to control the rate-limiting transition from the closed to open state as ADP and P_i_ are released (Reissmann et al., 2007). We have not examined this step of the catalytic cycle, but our measurements of the temperature dependence of the ATPase activities of apoCCTs strongly supports this hypothesis – the thermal coefficient of *G. gibberifrons* CCT is consistent with maintenance of structural flexibility at 4°C, whereas that for bovine CCT indicates a loss of flexibility at this temperature. We note, however, that the interdomain and intersubunit cooperativity intrinsic to the CCT folding cycle (Muñoz et al., 2011; Pereira et al., 2012) suggests that sequence changes in the intermediate and equatorial domains may also be involved in thermal adaptation of the *G. gibberifrons* chaperonin.

CCT binds quasi-native actin or tubulin to specific ring subunits via polar and electrostatic interactions (Ritco-Vonsovici and Willison, 2000; Llorca et al., 2001; Gómez-Puertas et al., 2004). Pucciarelli et al. have shown that the β and θ subunits of CCT from the Antarctic Bullhead notothen, *N. coriiceps*, contain multiple flexibility-enhancing amino acid substitutions (bulky/polar/charged residues in the CCT subunits of temperate fishes and mouse replaced by Ala or Gly in the psychrophilic fish subunits) in locations that should enable the conformational changes necessary for binding and release of CPs to occur at activation energies lower than those of mesophilic CCTs (Pucciarelli et al., 2006). We anticipate that a comprehensive survey of all *G. gibberifrons* CCT subunits will confirm this observation and that comparative structural analysis of *G. gibberifrons* and bovine CCTs (± bound CPs) will help to refine our understanding of the molecular interactions and catalytic mechanism of class II chaperonins. Conversely, we note that psychrophilic CPs also show evidence of increased structural mobility. Detrich et al. have shown that the α- and β-tubulins of Antarctic notothenioids have evolved more flexible M and N loops (Detrich et al., 2000), which likely strengthen interprotofilament interactions in microtubules at −1.9°C. Since the M and N loops of tubulins contribute importantly to binding to CCT (Gómez-Puertas et al., 2004), their increased flexibility in Antarctic fish tubulins should also enhance the CCT–CP interaction at low temperature. Together, these observations support the hypothesis that CCT and some CPs have co-evolved to maintain productive chaperonin-assisted folding reactions in a psychrothermal environment.

Comparison of enzymes obtained from psychrophilic and mesophilic organisms can be difficult due to differential thermal stability. As one increases the experimental temperature from the psychrophilic range (∼0 to 15°C) to the mesophilic (∼15–40°C), the anticipated exponential increase in the activity of the psychrophilic enzyme is likely to be compromised by an increased rate of denaturation. In this work, however, we found no evidence for denaturation-based decay of the ATPase activity of *G. gibberifrons* CCT at the low mesophilic temperature of 20°C. Similarly, we have shown that the brain and egg tubulins of Antarctic fishes assemble and disassemble reversibly at temperatures as high as 25°C with little evidence of denaturation (Detrich et al., 1989; Detrich et al., 1992; Detrich et al., 2000), whereas denaturation is clearly evident at 37°C. Thus, we suggest that 20–25°C may a thermal “sweet spot” for comparing the activities of psychrophilic and mesophilic enzymes.

Psychrophilic organisms, such as the Antarctic notothenioids, that have evolved in stable thermal environments over millions of years appear to be threatened by protein denaturation at both the upper and lower limits of their narrow thermal regimes. The very flexibility that maintains the functionality of their enzymes at physiological temperatures renders these proteins both heat labile *and* cold labile (Makhatadze and Privalov, 1995; Privalov, 1990; D'Amico et al., 2003). Decreased stability and unfolding at low temperature appear to be due to favorable changes in the contact free energy between nonpolar groups and water, such that peripheral penetration of water molecules weakens the hydrophobic effect and causes mechanical instability in the protein core (Lopez et al., 2008; Dias et al., 2010). The reality of cold-induced protein denaturation is well illustrated by the observation of elevated protein turnover in Antarctic fishes via ubiquitin-mediated proteasomal degradation (Todgham et al., 2007). Thus, CCT and the suite of chaperones that maintain protein homeostasis in Antarctic fishes provide novel opportunities for mechanistic analysis of cold denaturation using structural and biophysical strategies.

## MATERIALS AND METHODS

### Materials

Unless otherwise stated, reagents were purchased from Sigma–Aldrich (St Louis, MO, USA). Water was purified by use of Milli-Q systems (Millipore, Bedford, MA, USA).

### Collection of Antarctic fishes

Specimens of the Humphead notothen, *Gobionotothen gibberifrons* (Lönnberg), were collected by bottom trawls or via baited fish traps deployed from the *ARSV Laurence M. Gould* south of Low Island or west of Brabant Island in the Palmer Archipelago (April–June, 2008 and 2010). The fish were transported alive to Palmer Station, Antarctica, where they were maintained in seawater aquaria at −1.5 to 0°C. All procedures, including euthanasia, utilizing live vertebrate animals at Palmer Station, Antarctica, were reviewed and approved by Northeastern University's Institutional Animal Care and Use Committee.

### Purification of CCT from *G. gibberifrons* testis

All steps were carried out at −1 to +1°C (unless otherwise noted).

Immature testes (stages 2–3) from *G. gibberifrons* were homogenized in a Teflon-glass tissue grinder at a ratio of 1 g tissue per ml buffer H [40 mM HEPES-KOH (pH 7.35), 20 mM KCl, 2 mM EDTA] containing 1 mM DTT and 1 mM PMSF; one protease inhibitor tablet (cOmplete EDTA-free Protease Inhibitor Cocktail Tablets, Roche Diagnostics, Indianapolis, IN, USA) was added per 50 ml buffer H. The homogenate was centrifuged at 9600 × *g* for 1 h at 4°C, and the supernatant was recovered and centrifuged again at 105,000 × *g* for 1 h at 4°C. The second supernatant (designated testis extract) was flash frozen in liquid nitrogen and stored at −70°C.

After thawing, testis extracts were precipitated by addition of 30% (w/v) ammonium sulfate, and the suspension was centrifuged at 14,500 × *g* for 30 min at 4°C. The supernatant was recovered, ammonium sulfate was added to 50% (w/v), and the suspension was centrifuged again using the same parameters. The pellet was gently resuspended in a small volume of buffer A [50 mM Tris-HCl (pH 7.35), 150 mM NaCl, 5 mM MgCl_2_, 10% (v/v) glycerol] containing 1 mM DTT and one protease inhibitor cocktail tablet per 50 ml, and the suspension was dialyzed overnight against the same buffer without protease inhibitors. The dialyzed extract was loaded by use of a peristaltic pump onto two sequentially coupled 5-ml HiTrap Heparin Sepharose columns (GE Healthcare Bio-Sciences, Pittsburgh, PA, USA) pre-equilibrated with buffer A plus 450 mM NaCl. Bound proteins were eluted from the column by application of buffer A plus 600 mM NaCl, fractions of 1.5 ml were collected, and the protein compositions of the fractions were analyzed by SDS-PAGE (Laemmli, 1970); gels were stained with Coomassie Brilliant Blue R-250. CCT-containing fractions were pooled, the solution was made 80% (w/v) in ammonium sulfate, and the precipitated proteins were collected by centrifugation at 12,000 × *g* at 4°C. Supernatants were discarded, the pellets were resuspended in small volumes of buffer A and pooled, and the CCT-enriched sample was dialyzed against l× buffer A for at least 2 h with one buffer change. After dialysis, aliquots (1–1.5 ml) of the pool were loaded onto preformed sucrose gradients [10–50% (w/v)] in Beckman SW 28 open-top thick-wall polycarbonate centrifuge tubes, which were then centrifuged at 104,000 × *g* (28,000 rpm, r_av_ = 118.2 mm, Beckman SW-28 rotor) for 60 h at 4°C. Fractions (1 ml) were collected by lowering a glass needle, connected to a peristaltic pump, to the bottom of each tube. CCT-containing fractions, identified by SDS-PAGE, were pooled, flash frozen in liquid nitrogen, and stored at −70°C; some preparations were dialyzed against buffer A prior to flash freezing and storage. CCT was transported to our home institutions on dry ice.

The final step in the purification of *G. gibberifrons* CCT was size-exclusion chromatography of the sucrose-gradient-purified CCT on a Superose 6 10/300 GL column (GE Healthcare Bio-Sciences, Pittsburgh, PA, USA) equilibrated in buffer A and coupled to an ÄKTA Prime FPLC system (GE Healthcare Bio-Sciences, Pittsburgh, PA, USA) maintained at 6°C. The column was previously calibrated using the molecular size markers Dextran Blue (2000 kDa), thyroglobulin (670 kDa), ferritin (440 kDa), bovine serum albumin (67 kDa), and RNase (13.7 kDa).

### Separation of *G. gibberifrons* CCT subunits by HPLC

Purified *G. gibberifrons* CCT (280 µg) in buffer A was precipitated at 4°C by addition of trichloroacetic acid to 10%, and the suspension was centrifuged (15,000 × *g*, 4°C). The pellet was resuspended in 1 ml of 8 M urea, the suspension was diluted 8-fold with 0.1% trifluoroacetic acid, and the sample was centrifuged at 15,000 × *g* for 15 min (4°C). The supernatant was loaded (four injections of 2 ml each) on an XBridge BEH300 C4 Reversed-Phase HPLC column (2.1 × 50 mm; Waters Chromatografia S.A., Spain) coupled to an Ettan LC chromatography system (GE Healthcare Bio-Sciences, Pittsburgh, PA, USA). Bound proteins were eluted by application of dual gradients of acetonitrile [50–82% (v/v)] and trifluoroacetic acid [0.1–0.075% (v/v)] in 15 column volumes: solvent A = 50% acetonitrile, 0.1% trifluoroacetic acid; solvent B = 82% acetonitrile, 0.075% trifluoroacetic acid. Fractions (250 µl) were collected, solvent was evaporated by use of a Savant Speed-Vac (Thermo Fisher Scientific, Pittsburgh, PA, USA), and protein compositions of the peaks were analyzed by SDS-PAGE on 8.5% gels.

### Mass spectrometric analysis of *G. gibberifrons* CCT subunits

Protein bands containing CCT subunits were excised from Coomassie Blue-stained gels (see previous section), and automated in-gel protein digestion using modified porcine trypsin (sequencing grade; Promega, Madison, WI, USA) was performed on a Proteineer dp proteomics workstation (Bruker Daltonics, Bremen, Germany) according to established protocol (Shevchenko et al., 1996), with minor modifications. Peptide mass fingerprinting, MS/MS analysis, and peptide database searching were performed as described (Choi et al., 2013).

### Isoelectric focusing

Subunits of *G. gibberifrons* CCT, previously separated by HPLC and identified by mass spectrometry, were prepared for isoelectric focusing using an Agilent 3100 OFFGEL Fractionator and the OFFGEL pH 4–7 and 6–11 Kits (Agilent Technologies, Madrid, Spain) following the manufacturer's instructions. After drying in a Savant Speed-Vac (Thermo Fisher Scientific, Pittsburgh, PA, USA), each subunit sample was resuspended in 1 ml OFFGEL buffer containing 8 M urea, 2 M thiourea, 70 mM DTT, and 1.2% (v/v) ampholytes: pH 4–7 (Agilent Technologies, Madrid, Spain) were used with subunits α, β, γ, δ, ε, ζ, and θ, whereas pH 6–11 (GE Healthcare Bio-Sciences, Pittsburgh, PA, USA) was added to subunit η. Subunit samples were diluted to 3.6 ml by addition of OFFGEL fractionation buffer. Twenty-four aliquots of a subunit sample (150 µl each) were placed in the 24 wells of the OFFGEL tray, and focusing was performed on a 24-cm immobilized pH gradient (IPG) gel strip (linear pH gradient of 4–7 or 6.2–7.5, 50 µA) until 64 kVh was reached (∼48 h). After focusing, the 24 fractions were recovered and electrophoresed on an 8.5% SDS-PAGE gel. Each subunit pI was determined as the pH of the IPG well corresponding to highest subunit concentration.

The pIs of bovine CCT subunits were determined by focusing 300 µg of the purified complex on two IPG strips (pH 4–7 and 6.2–7.5) using the OFFGEL system described above. After SDS-PAGE of the 24 fractions from each strip, protein bands in the range 55–62 kDa were excised from each gel lane, and subunits were identified by mass spectrometry as described above. Subunit pIs were assigned by reference to the IPG pH gradient.

### Preparation of Antarctic fish client proteins (CPs)

Notothenioid CPs were obtained by expression of brain actin and tubulin cDNAs from two species, *Chaenocephalus aceratus* (Lönnberg) and *Notothenia coriiceps* (Richardson), that are closely related to *G. gibberifrons* (Eastman, 1993). *C. aceratus* β-actin (unpublished sequence, GenBank acc. no. KC594078) and *N. coriiceps* β1-tubulin (Detrich and Parker, 1993; acc. no. L08013), each cloned in pET 11a, were produced in *E. coli* as described (Gao et al., 1992) and modified (Pucciarelli et al., 2006). ^35^S-labeled CPs were expressed in medium containing 0.2 mCi of EasyTag™ L-[^35^S] methionine (NEG-709A, >1000 Ci/mmol, PerkinElmer, Waltham, MA, USA) and methionine-free amino acid mix. Unlabeled CPs were produced using complete amino acid mix. CPs were transferred to 7.5 M urea, 10 mM DTT, 20 mM Tris-HCl (pH 7.5) by gel filtration, and aliquots (5 mg/ml) were stored at −70°C.

### Preparation of bovine CCT and CPs

CCT from bovine testis was purified by the method of Martín-Benito et al. (Martín-Benito et al., 2002). Bovine cardiac actin (>99%; cat. no. AD99) and bovine brain tubulin (>99%; cat. no. TL238) were purchased from Cytoskeleton, Inc. (Denver, CO, USA).

### Structural characterization of CCT and CCT–CP complexes by EM

To compare the structures of apo- and holo-CCT particles from the psychrophilic fish to those of the mesophilic mammal, we generated two-dimensional average images by negative-stain EM. *G. gibberifrons* CCT was incubated in ATP-free binding buffer [50 mM Tris-HCl (pH 7.4), 500 mM NaCl, 5 mM MgCl_2_, 1 mM DTT, 10% (v/v) glycerol] (Cuellar et al., 2008) at 4°C in the absence or presence of homologous, denatured CPs (actin, tubulin), whereas bovine CCT was incubated with or without bovine CPs at 25°C. Denatured CPs from Antarctic fish or from the cow were diluted 100-fold into their respective CCTs to yield 10:1 molar ratios (1 µM CP, 0.1 µM CCT), and the samples were incubated for 5 min. Aliquots (5 µl) of each reaction were applied for 1 min to glow-discharged carbon-coated grids pre-cooled to the appropriate temperature. The samples were then stained for 1 min with 2% (w/v) uranyl acetate at the incubation temperature. Images were recorded at 0° tilt using a JEOL 1200EX-II electron microscope, operated at 100 kV, on Kodak SO-163 film at 20,000 × nominal magnification. Micrographs were digitized using a Zeiss SCAI scanner with a sampling window corresponding to 3.5 Å per pixel, and particles were automatically selected and classified using XMIPP software (Sorzano et al., 2009). Two-dimensional, reference-free average images of the end-on and side views of each CCT ± its CPs were averaged from ∼500–700 individual images (see legend to Fig. 3).

### CCT–CP binding and folding assays

To assess chaperonin–client protein affinities, denatured CPs from Antarctic fish or from the cow were combined with CCTs as described in the previous section, and the samples were incubated for 5 min at four temperatures between −4°C and +20°C in ATP-free binding buffer. Homologous binding (fish CCT–fish CP, etc.) experiments were performed in triplicate, whereas heterologous binding experiments (fish CCT–cow CP, etc.) were performed once, albeit with a very large sampling population. Samples from each reaction were prepared for negative-stain EM as described in the preceding section. Unless otherwise noted, 2000 end-on view CCT particles from each binding reaction were scored automatically as apo- or holo-CCT (determined by the absence or presence of a stain-excluding mass in the chaperonin cavity) using maximum-likelihood procedures (Scheres et al., 2005). After particle classification, the apparent affinity of binding was measured as the percentage of CCT particles containing bound substrate [(holo-CCT/holo-CCT + apo-CCT) × 100%].

The folding activity of *G. gibberifrons* CCT was assessed at 2°C by diluting denatured ^35^S-labeled *C. aceratus* β-actin 100-fold into chaperonin in binding buffer containing 1 mM ATP. At intervals, aliquots were withdrawn from the reaction, and the products (CCT–β-actin complex, folded actin) were analyzed on 4.5% non-denaturing polyacrylamide gels (Zabala and Cowan, 1992) run at the same temperature. Autoradiographs of the gels were scanned to quantify folded β-actin (Zabala and Cowan, 1992).

### ATPase assays

Rates of ATP hydrolysis by apoCCT from *G. gibberifrons* or from the cow were measured spectrophotometrically in a buffer containing a coupled-enzyme, ATP-regenerating system (Sot et al., 2002). Reaction mixtures (50 mM Tris-HCl, 10 mM MgCl_2_, 100 mM KCl, 0.2 mM NADH, 2 mM phosphoenolpyruvate, 15 µg/ml pyruvate kinase, and 30 µg/ml lactate dehydrogenase, pH 7.5) were pre-equilibrated at the desired temperature (either 4 or 20°C) for 10 min in the thermostated cuvettes of a Shimadzu CPS-240A spectrophotometer. ATP (2 mM final concentration) was added to each cuvette, and the assay mixtures were incubated isothermally for 2 min. Finally, CCT (0.38 µM final concentration) was added, and the decrease in absorbance at 340 nm was followed for intervals up to 120 min. Triplicate assays were performed at each temperature. We verified that the activities of the coupling enzymes were sufficiently high at both temperatures such that the ATPase activity of CCT was the limiting factor controlling the oxidation of NADH.
